# Perceived helpfulness of various sources of help for 5 mental illnesses

**DOI:** 10.1192/j.eurpsy.2024.448

**Published:** 2024-08-27

**Authors:** S. Shahwan, E. H. Tay, S. A. Chong, M. Subramaniam

**Affiliations:** Research, IMH, Singapore, Singapore

## Abstract

**Introduction:**

1 in 8 people worldwide live with a mental illness (MI). This is expected to rise with increasing societal pressures. Despite the availability of evidence-based treatments, MIs remain undertreated. In Singapore, efforts such as the ‘It’s OK to Reach Out’ campaign was launched to encourage help-seeking. Help-seeking behavior is complex; determined by an interplay of factors including perceptions towards help sources. As seeking ineffective sources contributes to unmet needs, understanding beliefs towards various sources of help is vital.

**Objectives:**

The study aims to examine perceived helpfulness of various sources of help for 5 mental illnesses and changes in perceptions towards them over time.

**Methods:**

The Mind Matters 2023 (M2) is an ongoing nationwide survey of mental health literacy among Singapore residents aged 18-65 years. Analysis is based on a preliminary sample (N=2500). Interviewers read a vignette depicting 1 of 5 randomly assigned MIs- depression (DP), schizophrenia (SZ), obsessive-compulsive disorder (OCD), alcohol abuse (AA) or dementia (DT). Respondents were asked to rate whether 10 Professional/Informal sources and 12 Actions were ‘helpful, ‘harmful’ or ‘neither’ for the person in the vignette. Frequencies of helpful ratings were compared with the first Mind Matters study (M1) conducted in 2015 (N=3006).

**Results:**

In M2, seeing a psychiatrist, psychologist and counsellor were rated helpful most frequently (79%-96%) while seeking traditional medicine and religious advisors were rated the least (12%-60%) across the vignettes, except for DT where doctor and close family (81%-85%) replaced psychologist and counsellor (66%-70%). Compared to M1, phone counselling saw an increase in helpfulness rating across all vignettes (p<.05) except AA. For Actions, reading about how others dealt with similar problems was rated helpful most frequently (76%-89%) while dealing with problems on one’s own was rated the least across all vignettes (3%-11%) in M2. Compared to M1, being more social saw an increase in helpfulness (p<.001) rating for DP but a decrease for AA (p<.001). Admission to an institution was associated with a decrease in helpfulness rating for DP (p=.006) and OCD (p=.04) but increase for AA (p=.03).

**Conclusions:**

The findings suggest recognition that MIs would be helped by professionals and self-reliance is ineffective to address these problems. Increased perceived helpfulness of telephone counselling was promising as studies have shown high client satisfaction coupled with its potential in reducing some barriers to care. Differences in directional changes in helpfulness rating for institutional care and socialising for DP and AA may represent understanding of the importance of behavioral activation and stimulus control for these MIs respectively. Literacy regarding help-seeking sources has improved in Singapore over the last 8 years which may translate into increments in seeking appropriate care.

**Disclosure of Interest:**

None Declared

